# Developing Lotka–Volterra Based Models to Describe *Bdellovibrio* Predation in a Batch and Chemostat Experimental System

**DOI:** 10.1111/1758-2229.70141

**Published:** 2025-07-06

**Authors:** Ayo Ogundero, Stephanie Connelly, William T. Sloan

**Affiliations:** ^1^ Infrastructure and Environment, School of Engineering University of Glasgow UK

**Keywords:** *Bdellovibrio*, flow cytometry, Holling, Lotka–Volterra, mathematical model, predator–prey oscillations, predatory bacteria

## Abstract

The application of *Bdellovibrio* predatory bacteria as an antibiotic alternative is hindered by the lack of experimentally validated models. To address this, we use flow cytometry as a high‐throughput method to accurately quantify *Bdellovibrio bacterivorous* and *Pseudomonas* sp. prey growth in batch culture, enabling the determination of key growth parameters. We then develop Lotka–Volterra based predator–prey mathematical models with Holling type II and Holling type III dynamics, incorporating glucose as the prey substrate. We conduct experiments in batch and chemostat cultures to evaluate the ability of the model to predict *B. bacterivorous* predation. In batch systems, *B. bacteriovorus* dynamics can be captured by the Holling type III numerical response (distance correlation = 0.999), which supports the hypothesis of premature prey lysis at high predator–prey ratios. Using chemostat simulations, we identify parameter regimes leading to predator washout, stable coexistence, or predator–prey oscillations. We evaluate this by inducing an experimental realisation of sustained predator–prey oscillations in a chemostat. This is a key phenomenon necessary for self‐sustaining biocontrol. Our findings provide a quantitative foundation for optimising *B. bacteriovorus* applications as a biocontrol agent across diverse fields, including clinical therapy, agriculture, and water treatment.

## Graphical abstract

Using flow cytometry, we obtain key growth parameters of *Pseudomonas* and *Bdellovibrio* predatory bacteria which we use to develop Lotka–Volterra mathematical based models to predict the change in predator, prey, and glucose in batch and chemostat systems. We simulate the regions of parameter space to yield different characteristic dynamics in a chemostat including predator–prey oscillations. We validate this by inducing one of the first experimental realisations of Bdellovibrio predator–prey oscillations in a chemostat experiment.

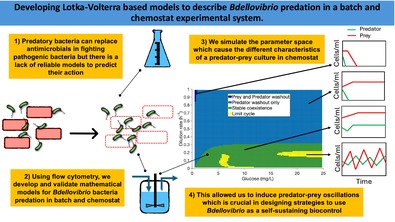

## 1 Introduction

*Bdellovibrio bacteriovorus* is a predatory bacterium with potential as a biocontrol agent against biofilms, offering an alternative to chemical treatments in various engineered systems (Chen et al. 2010; Dwidar et al. 2012; Klein and Casida 1967). For instance, its use in drinking water membrane filters to prevent biofilm growth requires a clearer understanding of its population dynamics, including how environmental factors such as prey density and dilution rate influence long‐term predation efficiency.

Due to the complexity of microbial interactions and fluctuating environmental conditions, empirical trial and error testing alone is insufficient for predicting predation outcomes. Mathematical models provide a valuable framework for simplifying and analysing these dynamics. By abstracting key processes such as growth, predation, and nutrient limitation, they offer a systematic approach to predict microbial behaviour and inform biocontrol strategies (Li et al. 2007).

### 1.1 The Lotka–Volterra Predator–Prey Model

Classical models such as Lotka–Volterra (LV) provide a foundational understanding of predator–prey interactions, capturing cyclic dynamics through differential equations (Berryman 1992). The LV model reflects two core principles of population ecology: exponential growth in the absence of limiting factors and cyclical population fluctuations driven by trophic interactions (Blasius et al. 2020; Turchin 2001). However, as Turchin (2001) outlines, many ecological systems fail to exhibit the idealised oscillations predicted by LV models largely because of simplifying assumptions such as unlimited prey growth and linear predator consumption, which rarely hold in real biological systems.

Nonetheless, empirical studies have shown stable coexistence and oscillations between *B. bacteriovorus* and prey under specific conditions (Varon et al. 1984), yet long‐term biofilm suppression likely depends on maintaining oscillatory predator–prey dynamics rather than stable co‐existence alone. These sustained oscillations can support predator persistence and improve system adaptability, especially in decentralised systems like membrane filters with limited monitoring. Thus, it is important to develop models that can distinctly and efficiently allow for the induction of oscillations and the investigation of all the potential outcomes of a predator–prey system.

To better adhere to foundational principles of population ecology, refinements to LV models should incorporate biological constraints such as nutrient‐limited prey growth and saturating predator responses (Turchin 2001). One key extension for the former is the Monod model, which describes microbial growth as a function of substrate concentration. When all other nutrients are saturated and non‐limiting, but one key nutrient is too low, the growth rate is proportional to the concentration of the limited nutrient. At optimal concentrations the growth rate will be at the maximum value (Bren et al. 2013). However, when the nutrient concentrations increase to too high a value it can inhibit growth, causing the growth rate to fall (Johnson and Stokes 1965). Although Monod (1949) originally presented this relationship as an empirical steady‐state function rather than a dynamic kinetic model, it is widely used in microbial ecology to represent growth‐limiting conditions. The Monod function mirrors Michaelis–Menten kinetics in form and is commonly embedded in modern time‐dependent models to simulate growth dynamics (Canale 1969).

Similarly, predator responses are better captured by Holling functional forms. The Holling type II model introduces predator saturation via a prey‐dependent consumption rate, and has been successfully applied to both protists (DeLong and Vasseur 2012) and *Bdellovibrio* (Summers and Kreft 2022). These models better reflect biological limits, such as the time predators spend handling prey. For *Bdellovibrio*, this includes the intracellular replication phase post prey‐invasion, during which time the predator cannot interact with other bacteria and progeny bacteria are waiting to be released (Sockett 2009).

### 1.2 Modelling Predator–Prey Growth in Batch Cultures

We can extend the LV model with Monod and Holling Type II functions to allow for substrate‐limited prey growth and saturating predator feeding, respectively. When describing predator–prey growth in batch cultures, this results in the set of equations:

(1) $$\frac{\mathrm{dS}}{\mathrm{dt}}=-\left(\frac{\mu_{X}S}{K_{S}+S\;}\right){\frac{X}{\zeta }}_{S}$$

(2) $$\frac{\mathrm{dX}}{\mathrm{dt}}=\left(\frac{\mu_{X}S}{K_{S}+S\;}\right)X-\left(\frac{\mu_{Y}X}{K_{X}+X\;}\right){\frac{Y}{\zeta }}_{X}$$

(3) $$\frac{\mathrm{dY}}{\mathrm{dt}}=\left(\frac{\mu_{Y}X}{K_{X}+X\;}\right)Y$$

Here, Equation (1) describes the limiting substrate utilisation $\left(dS/dt\right)$, where S is the growth limiting substrate concentration (mg/L). $\mu_{X}$ (h^−1^) is the maximum specific growth rate of the prey and $K_{S}$ (mg/L) is the substrate half‐saturation constant. The half‐saturation constant is the concentration of growth rate‐limiting nutrient that supports half the maximum specific growth rate. $\zeta_{S}$ (cells/mg of substrate) is the prey yield, which is the number of new prey cells produced per mass of substrate consumed and is assumed to be constant.

The half‐saturation constant used in the Monod law is versatile and has the ability to simplify processes that may involve more than one enzyme, which may make it suitable to describe *B. bacteriovorus* growth on prey bacteria as the ‘substrate’ (Merkel et al. 1996; Wilkinson 2006). It is expected that when the substrate concentration is high enough and saturating, the maximal growth rate is achieved and the growth rate of the prey is constant (Owens and Legan 1987).

The rate of change of prey $\left(dX/dT\right)$ in Equation (2) is a function of growth on the substrate and the death by predation as determined by the predator growth and the predator yield $\zeta_{X}$ (predator cell/prey cell) which is the number of new predator cells produced per prey consumed. The rate of change of the predator $\left(dY/dT\right)$ in Equation (3) takes into account the prey concentration (*X*, cells/mL) which in this case is the growth‐limiting ‘substrate’, the maximum specific growth rate of the predator $\mu_{Y}$ (h^−1^) and the prey half‐saturation constant (*K*_*X*_, cells/mL). The prey half‐saturation constant is the concentration of prey that supports half the maximum specific growth rate of the predator. It is assumed that *B. bacteriovorus* does not grow or directly interact with the substrate and, as above, the predator does not grow in the absence of prey (Lambert and Sockett 2008).

While the Holling type II model assumes saturating predation, it does not account for reduced predator efficiency at low prey densities. In contrast, the Holling type III model introduces a sigmoidal response, where predation is inefficient at low prey density and improves with increasing prey availability. Type III functional responses may arise from a variety of mechanisms and are typical of predators which show some kind of learning behaviour such as developing a more specialised way of killing prey (DeLong 2021; Morozov and Petrovskii 2013). It is also used to describe predators responding to increased chemical stimuli from prey or in cases where a larger amount of energy is expended in finding the prey than is returned (Jost et al. 1973; Real 1977). These cases are not definitive with *B. bacteriovorus* and as such the Holling type III functional response has not previously been shown.

However, many studies have described the random collision‐based hunting method of *B. bacteriovorus* (Jurkevitch 2006; Wilkinson 2001). Assuming a lack of significant and specific searching for prey when the system is well mixed suggests that, especially at low prey densities, there is an increased chance of the *B. bacteriovorus* cell attaching to a prey cell that is already being handled by another *B. bacteriovorus* cell, causing it to eventually detach. Furthermore, when multiple *B. bacteriovorus* cells attach to a single prey cell, it can cause the prey cell to burst prematurely before successful invasion, resulting in energetic costs without reproductive return (Im et al. 2014). In practice, this inefficiency at low prey densities may be overcome as prey density increases, improving the likelihood of successful encounters and invasion. This dynamic can generate a sigmoidal relationship between predator growth rate and prey density—characteristic of a Holling type III numerical response.

This relationship can be displayed by modifying Equations ((1), (2), (3)) to the following:

(4) $$\frac{\mathrm{dS}}{\mathrm{dt}}=-\left(\frac{\mu_{X}S}{K_{S}+S\;}\right){\frac{X}{\zeta }}_{S}$$

(5) $$\frac{\mathrm{dX}}{\mathrm{dt}}=\left(\frac{\mu_{X}S}{K_{S}+S\;}\right)X-\left(\frac{\mu_{Y}X^{2}\;}{K_{X}+X^{2}\;}\right){\frac{Y}{\zeta }}_{X}$$

(6) $$\frac{\mathrm{dY}}{\mathrm{dt}}=\left(\frac{\mu_{Y}X^{2}}{K_{X}+X^{2}\;}\right)Y$$

This equation involves squaring the value of *X* to take into account the incremental amount of prey needed to increase the efficiency of predation. This change allows exploration of oscillatory and threshold behaviours under both functional responses.

### 1.3 Modelling Predator–Prey Growth in Continuous Cultures

Continuous culture allows the control of fluid (bacteria or substrate) input into the system as determined by the concentration present in a reservoir system and the precise control of dilution rate (Senn et al. 1994). Bacterial growth in chemostats is commonly investigated in engineering, through the use of models or as an experimental system (Canale et al. 1973; Rana et al. 2015). In a chemostat, the predator/prey/substrate system in Equations ((1), (2), (3)) can now be described as:

(7) $$\frac{\mathrm{dS}}{\mathrm{dt}}=D\left(S_{0}-S\right)-{\frac{1}{\zeta }}_{S}\left(\frac{\mu_{X}S}{K_{S}+S\;}\right)X$$

(8) $$\frac{\mathrm{dX}}{\mathrm{dt}}=-\mathrm{DX}+\left(\frac{\mu_{X}S}{K_{S}+S\;}\right)X-\frac{1}{\zeta_{X}}\left(\frac{\mu_{Y}X}{K_{X}+X}\right)Y$$

(9) $$\frac{\mathrm{dY}}{\mathrm{dt}}=-\mathrm{DY}+\left(\frac{\mu_{Y}X}{K_{X}+X}\right)Y$$

where *S*_0_ represents the substrate concentration (mg/L) in the inflow and *D* the dilution rate (h^−1^).

As before we can square the value of X in the predation term to transform Equations ((7), (8), (9)) and reflect the Holling type III response:

(10) $$\frac{\mathrm{dS}}{\mathrm{dt}}=D\left(S_{0}-S\right)-{\frac{1}{\zeta }}_{S}\left(\frac{\mu_{X}S}{K_{S}+S\;}\right)X$$

(11) $$\frac{\mathrm{dX}}{\mathrm{dt}}=-\mathrm{DX}+\left(\frac{\mu_{X}S}{K_{S}+S\;}\right)X-\frac{1}{\zeta_{X}}\left(\frac{\mu_{Y}X^{2}}{K_{X}+X^{2}}\right)Y$$

(12) $$\frac{\mathrm{dY}}{\mathrm{dt}}=-\mathrm{DY}+\left(\frac{\mu_{Y}X^{2}}{K_{X}+X^{2}}\right)Y$$

To test whether the basic tenets of predator–prey models hold, predator–prey dynamics are explored in this study, in the simple batch (Equations (1), (2), (3) for Holling type II and Equations (4), (5), (6) for Holling type III) and chemostat systems (Equations (7), (8), (9) for Holling type II and Equations (10), (11), (12) for Holling type III) that have been described above. The rationale used here is initially to derive parameters for the batch models from careful experimentation in highly simplified scenarios. Then to use the parameters to simulate more complex dynamics of predator–prey interactions in a chemostat.

The aim then is to validate the results of the simulations using further careful experimentation. Thus, the modelling is being used to guide experimentation. In an engineering application such as a drinking water membrane filter it is desirable to then design a system where there is no requirement for repeated inoculations of the predator, instead they remain in the system while continuing to keep the, potentially biofouling, prey at bay. Thus, maintaining predator–prey oscillations or cycles is of particular interest. Overall, the modelling should elucidate the key parameter regime that will deliver predator–prey cycling in the laboratory experiment.

## 2 Materials and Methods

### 2.1 The Predator and the Prey

The wild‐type *Bdellovibrio bacteriovorus* strain HD100 (DSM no. 50701) was used throughout this study and was grown by predation on *Pseudomonas* sp. (DSM no. 50906) using standard culturing methods (Herencias et al. 2017). *Pseudomonas* sp. was grown overnight in LB broth at 30°C, then resuspended in Ca/Mg‐HEPES buffer (25 mM HEPES, 2 mM CaCl_2_, 3 mM MgCl_2_, pH 7.6) to OD600 = 10. *B. bacteriovorus* was then grown on this prey suspension in DN broth for 24 h at 30°C, followed by two subcultures at 24‐h intervals in Ca/Mg‐HEPES. Cultures were filtered through 0.45 μm syringe filters (Fisherbrand 15216869) to remove prey cells and obtain predator‐only filtrate.

### 2.2 Identifying the Batch Model Parameters

To determine Monod parameters describing prey growth as a function of glucose concentration, overnight *Pseudomonas* sp. cultures were harvested and standardised to 10^5^ cells/mL. Aliquots were introduced into 500 mL flasks containing 100 mL of minimal medium with 0, 0.5, 1, 2.5, 5, or 20 mg/L glucose. Cultures were incubated for 16 h at 30°C with shaking at 200 rpm. For prey growth analysis, samples were fixed 1:1 in glutaraldehyde, stored at 4°C, and analysed by flow cytometry within 3 days. For glucose analysis, aliquots were taken every 2 h, filtered, frozen at −20°C, and assayed using a Sigma glucose kit.

To determine prey–predator dynamics, *B. bacteriovorus* filtrate (10^5^ cells/mL) was inoculated into 50 mL cultures of *Pseudomonas* sp. with varying concentrations (10^6^–10^9^ cells/mL) in Ca/Mg‐HEPES buffer. Cultures were incubated at 30°C for 72 h. Prey and predator dynamics were monitored by glutaraldehyde fixation and flow cytometry.

### 2.3 Model Implementation

The numerical solutions of the batch (Equations (1), (2), (3) for Holling type II and Equations (4), (5), (6) for Holling type III) and chemostat cultures (Equations (7), (8), (9) for Holling type II and Equations (10), (11), (12) for Holling type III) were implemented in MATLAB using the parameters obtained in the batch culture parameter assays. The codes used the robust, ordinary differential equation package ODE45. The codes are included in Appendix S1.

### 2.4 Batch Predator–Prey Simulations and Experiments

**FIGURE 1:**
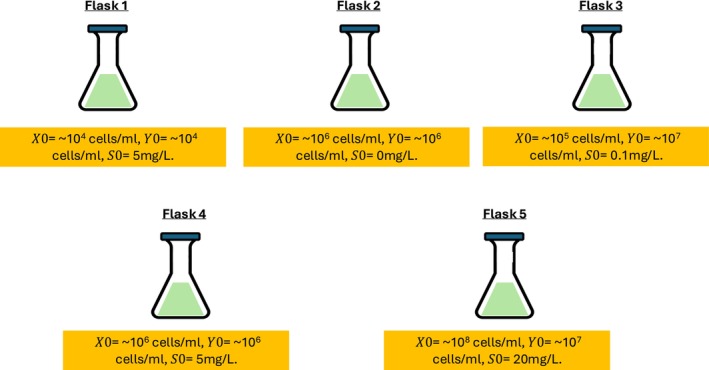
Schematic diagram represents the flasks for the batch culture experiments. Initial conditions with different initial prey (*X*_0_), predator (*Y*_0_), and glucose substrate (*S*_0_), concentrations that were randomly selected are shown.

Batch model simulations (Equations (1), (2), (3), (4), (5), (6)) were conducted for five flasks with distinct initial conditions, selected to capture a range of behaviours such as differences in the speed and degree of change of the prey and predator population (Figure 1). Matching experiments were run in 250 mL flasks with 50 mL of minimal medium with various combinations of glucose, prey, and predator concentrations. Samples for glucose, prey, and predator were collected over 72 h.

### 2.5 Chemostat Simulations

To explore dynamic outcomes under varying conditions, the chemostat model (Equations (7), (8), (9) for Holling type II and Equations (10), (11), (12) for Holling type III) was run in MATLAB (code is included in Appendix S1) for every combination of *S*_0_, influent glucose concentration, and *D*, dilution rate, selected from vectors with 100 equal increments in the ranged (0.5, 25) mg/L and (0.01, 1) h^−1^. The dynamics of predator and prey abundances for each combination were simulated for 40 hydraulic retention times, 40/*D* h. If the simulated abundances of predator and prey at the end of the simulation were zero, then the dynamics were classified as ‘washout’. If the prey abundance remained constant over the last 20 retention times and the predator abundance dropped to zero, this was classified as predator washout. If both abundances were constant, this was classified as stable co‐existence. If the abundances of predator and prey over the last 20 retention times crossed their mean values more than four times, then the solution was deemed to be oscillating and, thus, exhibiting a ‘limit cycle’.

### 2.6 Chemostat Experiment Run 1

To evaluate the model's predictive accuracy, a chemostat was operated for 168 h using the Applikon my‐Control bioreactor. *Pseudomonas* sp. and *B. bacteriovorus* cultures were used to inoculate the bioreactor chamber, and minimal medium (HEPES buffer with 2 mM calcium chloride, 3 mM magnesium chloride, 0.05% casein hydrolysate, and 25 mg/L glucose) was continuously supplied. Inlet and outlet flow were regulated at 0.17 h^−1^ by peristaltic pumps. Samples of prey, predator, and substrate were taken from the effluent throughout.

### 2.7 Chemostat Experiment Run 2

To investigate biofilm growth and explain discrepancies observed in Run 1, the chemostat was operated again for 72 h with the addition of sterile glass slides. At the end, biofilm grown on the glass slides was reconstituted into PBS via sonication and quantified by flow cytometry. Effluent samples were also analysed.

### 2.8 Flow Cytometry

Bacterial concentrations were determined by flow cytometry (Ogundero et al. 2022). Samples were fixed in 1% glutaraldehyde and diluted in 0.22 μm‐filtered DI water to keep event rates < 600/s. Total counts were obtained by staining with SYBR Green I (10 μL/mL), with stock dye diluted 1:100 in 1 mM EDTA. Gating was used to distinguish *B. bacteriovorus* and *Pseudomonas* sp. from each other and from debris using negative controls including DI water, HEPES buffer, and filtered co‐cultures.

### 2.9 Glucose Measurement

To measure the amount of glucose in a sample, filtered samples were frozen at −20°C and assayed using the Sigma Glucose assay kit (MAK263‐1KT) per manufacturer's instructions. Absorbance was measured at 570 nm in microwell plates.

### 2.10 Statistical Analysis of Model Performance

Model accuracy was assessed using root mean square error (RMSE) and distance correlation (dCor) in RStudio. These metrics were calculated for glucose, prey, and predator concentrations, with raw values for glucose and log_10_‐transformed cell counts for comparability.

Specifically, RMSE is used to quantify the absolute error between predicted and observed data. It is expressed in the same units as the dependent variable and a lower RMSE indicates less systematic error in the model (Chai and Draxler 2014). Distance correlation was used to test how well the trends match. It is expressed as a value from 0 to 1, with values closer to 1 indicating that the model fits the general biological pattern (Székely and Rizzo 2009). To assess the statistical significance of the computed dCor values, a permutation test with 10,000 randomisations is performed. While the focus was on capturing qualitative trends in predator–prey interactions, these additional metrics help quantify the agreement between model predictions and experimental data.

## 3 Results

### 3.1 Batch Model Parameter Calculation

To measure the growth of prey more accurately and reliably capture the growth parameters that are critical to the batch model, flow cytometry (see Table S1 for raw bacterial counts) is used to measure the batch culture growth of *Pseudomonas* sp. on minimal media supplemented with a range of glucose concentrations from 0 to 20 mg/L.

**FIGURE 2:**
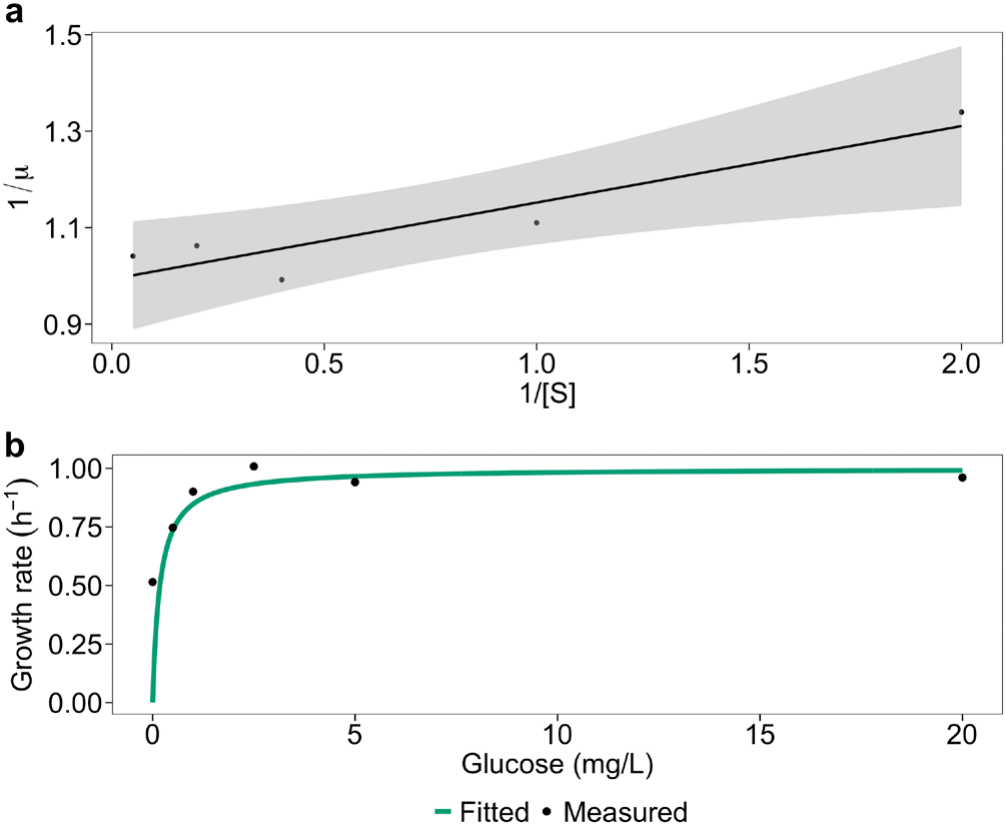
Relationship of *Pseudomonas* sp. growth with glucose. (a) Lineweaver–Burk plot with confidence intervals (grey) of the relationship between μ, the specific growth rate (h^−1^) of *Pseudomonas* sp. vs. S, the initial concentration of glucose (mg/L). Linear regression trendline equation: *Y* = 0.1586*x* + 0.9933, *R* ^2^ = 0.8675. (b) The half‐saturation constant (0.18 mg/L) and maximum growth rates (1.0 h^−1^) are calculated to model the relationship using the Monod equation. Evaluation of model performance: RMSE = 0.044 h^−1^, dCor = 0.942 (*p* = 0.043). Raw bacterial counts and the growth rate calculations are provided in Tables S1 and S2, respectively.

To determine the specific growth rate, a regression is fit to the logarithmic cell counts during the exponential growth phase, with the slope of the line representing the growth rate. A Lineweaver–Burk plot of the growth rate under different concentrations of glucose is then plotted. This plot is a linearised forms of the Monod equation (Figure 2a) and so by fitting a linear regression trendline, the intercept and the slope is obtained to determine the value of the maximum growth rate (1.0 h^−1^) and the half‐saturation constant (0.18 mg/L). Here, the maximum growth rate is: 1/the intercept and the half‐saturation constant is the slope × the maximum growth rate (Owens and Legan 1987). With this the Monod equation is used to predict the relationship between bacterial growth rate and the concentration of glucose.

*Pseudomonas* sp. growth on glucose fits Monod theory well (Figure 2b). As the initial glucose concentration increases, *Pseudomonas* sp. growth rate increases exponentially up to approximately 2.5 mg/L, where the growth rate levels off.

The model predicts no growth in the absence of glucose (Figure 2b), whereas experimental data show that *Pseudomonas* sp. grows at a rate of 0.51 h^−1^. However, at all other glucose concentrations, the model closely aligns with observed measurements, as reflected in a low overall RMSE (0.044 h^−1^). Additionally, a significantly high distance correlation (dCor = 0.942, *p* < 0.05) indicates that, particularly at higher glucose concentrations, the model accurately captures the trend in growth dynamics.

A growth curve is measured of *B. bacteriovorus* grown on Ca/Mg‐HEPES buffer inoculated with different concentrations of *Pseudomonas* sp. ranging from a severely limiting level (0 cells/mL), to saturating level (10^9^ cells/mL).

**FIGURE 3:**
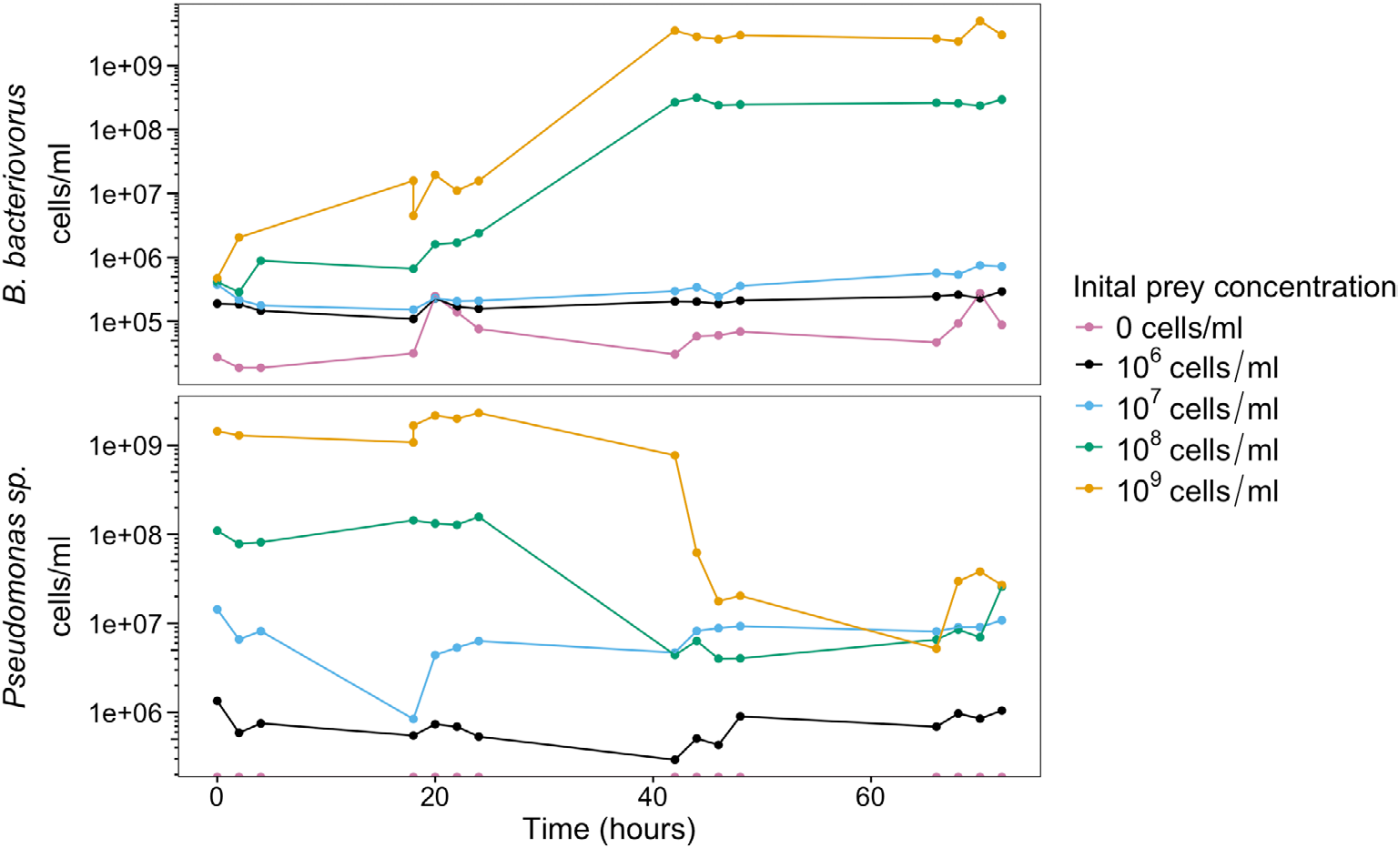
Growth curve of *B. bacteriovorus* and *Pseudomonas* sp. (cells/mL) population during batch culture with different initial prey concentrations. Raw bacterial counts are provided in Table S3.

Figure 3 shows a typical growth in a batch culture of *B. bacteriovorus* and *Pseudomonas* sp. For the 72 h there is little to no change in the concentrations of prey and the predator when the initial *Pseudomonas* sp. concentration is 10^7^ cells/mL or lower. However, when the prey concentration is increased to 10^8^ cells/mL the predator population increases slowly for the first 24 h before rapidly increasing and then levelling off at around 44 h. Simultaneously, the prey population shows little change at high initial prey concentrations for the first 24 h before rapidly decreasing as a result of predation. The prey population then levels off after 48 h.

**FIGURE 4:**
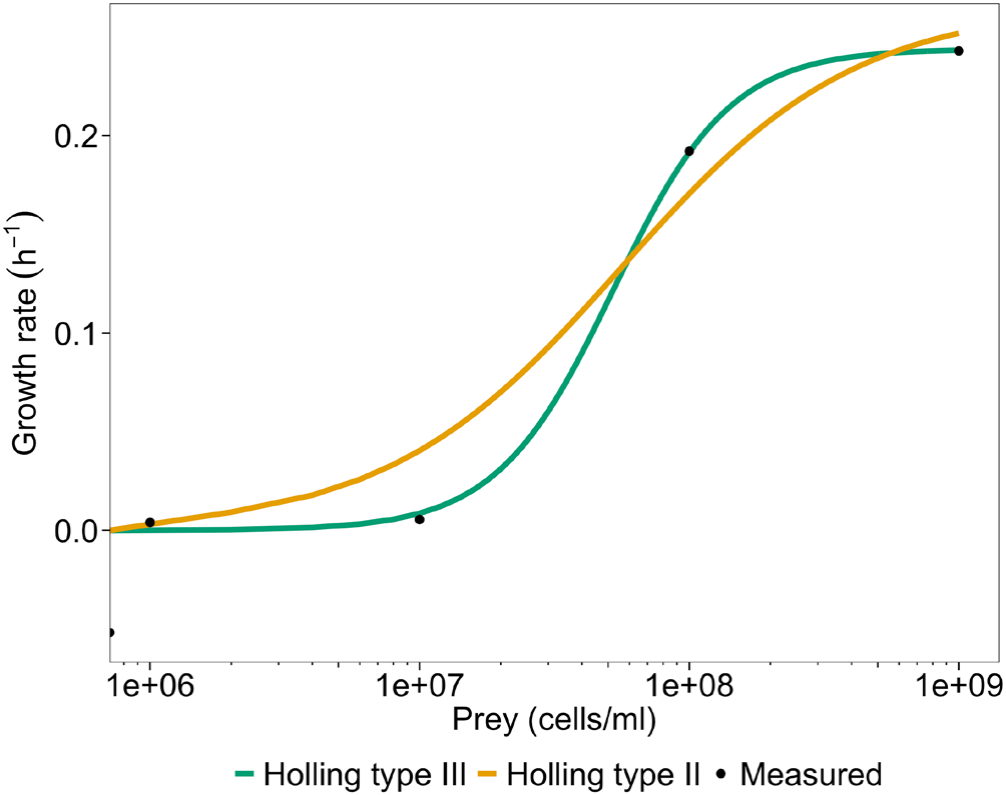
Specific growth rate (h^−1^) of *B. bacteriovorus* vs. the initial concentration of *Pseudomonas* sp. prey (cells/mL) in batch culture. The half‐saturation constant and maximum growth rates are calculated to model the relationship using the Holling type II equation (0.266 h^−1^ and 5.59 × 10^7^ prey cells/mL) and Holling type III equation (0.244 h^−1^ and 2.74 × 10^15^ cells^2^/mL^2^). Evaluation of model performance: Holling type II: RMSE = 0.024 h^−1^, dCor = 0.983 (*p* = 0.046). Holling type III: RMSE = 0.003 h^−1^, dCor = 0.999 (*p* = 0.044). Growth rate calculations are provided in Table S4.

As in Figure 2, with the prey dynamics, the *B. bacteriovorus* growth curve data is used to plot the *B. bacteriovorus* specific growth rate as a function of *Pseudomonas* sp. prey cell density (Figure 4). This concurs with the observations of Figure 3; the predator growth rate shows no increase at low concentrations of prey but when the prey concentration increases the predator growth rate rapidly increases. The intercept and slope of a Lineweaver–Burk plot is used as previously to determine the maximum growth rate and half‐saturation constant when using both Holling type II (0.266 h^−1^ and 5.59 × 10^7^ prey cells/mL respectively) and the Holling type III equations (0.244 h^−1^ and 2.74 × 10^15^ cells^2^/mL^2^ respectively). These model equations are used to predict the relationship between the predator specific growth rate and prey density.

Though both the Holling type II (dCor = 0.983) and Holling type III (dCor = 0.999) equations can capture the trend of growth rate in response to prey concentration well (*p*‐value < 0.05), the observed growth is more accurately predicted by a Holling type III numerical response rather than Holling type II, which overestimates the increase in growth rate when increasing prey concentration from 10^6^ to 10^7^ cells/mL. This is confirmed by RMSE, which gives a value of 0.024 h^−1^ for the Holling type II model but 0.003 h^−1^ for the Holling type III model. Neither the Holling type II nor Holling type III equations are designed to predict the decrease in predator population observed in the absence of prey, though this could be resolved with the addition of a predator mortality rate constant (Wilkinson 2006).

The batch culture experiments above allowed us to estimate the parameter values for the predator–prey growth kinetics (maximum growth rates and half‐saturation constants); these are summarised and presented in Table 1.

**TABLE 1:** Kinetic parameters used in model Equations ((4), (5), (6), (7), (8), (9)).

Kinetic parameter	Holling type II values (Equations (1), (2), (3) and (7), (8), (9))	Holling type III values (Equations (4), (5), (6) and (10), (11), (12))
Maximum prey growth rate	1.0 h^−1^	1.0 h^−1^
Maximum predator growth rate	0.266 h^−1^	0.244 h^−1^
Predator yield	1.95 *B. bacteriovorus* cells/*Pseudomonas* sp. cell	1.95 *B. bacteriovorus* cells/*Pseudomonas* sp. cell
Prey yield	3.1 × 10^7^ cells/mg of glucose	3.1 × 10^7^ cells/mg of glucose
Glucose saturation constant	0.18 mg/L	0.18 mg/L
Prey saturation constant	5.59 × 10^7^ cells/mL	2.74 × 10^15^ cells^2^/mL^2^

### 3.2 Batch Validation/Fitting

**FIGURE 5:**
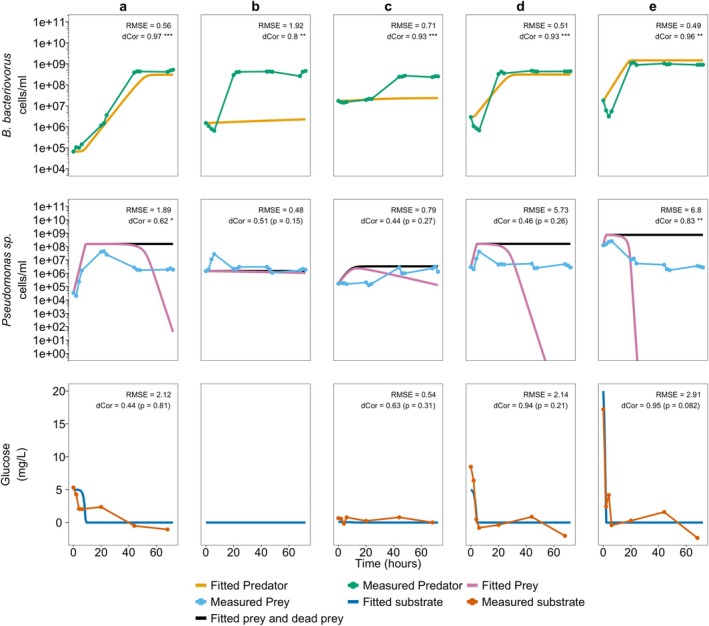
Transient curve of Holling type II (Equations (1), (2), (3)) fitted and experimentally observed *B. bacteriovorus* and *Pseudomonas* sp. (cells/mL) population and glucose (mg/L) during five different batch cultures (a–e) with different initial prey, predator, and glucose substrate concentrations. (a) *X*_0_ = ~10^4^ cells/mL, *Y*_0_ = ~10^4^ cells/mL, *S*_0_ = 5 mg/L. (b) *X*_0_ = ~10^6^ cells/mL, *Y*_0_ = ~10^6^ cells/mL, *S*_0_ = 0 mg/L. (c) *X*_0_ = ~10^5^ cells/mL, *Y*_0_ = ~10^7^ cells/mL, *S*_0_ = 0.1 mg/L. (d) *X*_0_ = ~10^6^ cells/mL, *Y*_0_ = ~10^6^ cells/mL, *S*_0_ = 5 mg/L. (e) *X*_0_ = ~10^8^ cells/mL, *Y*_0_ = ~10^7^ cells/mL, *S*_0_ = 20 mg/L. Raw observed bacterial counts and the model predicted counts are provided in Table S5. Root mean square error (RMSE) and distance correlation (dCor) values for the comparison between the batch model fitted and observed experimental values are shown in the plot. Analysis for the *Pseudomonas* sp. and *B. bacteriovorus* used the log_10_ transformed values. RMSE is expressed in the log_10_ (cells/mL). *Statistical significance of dCor: *p < 0.05, **p < 0.01, and ***p < 0.001.

**FIGURE 6:**
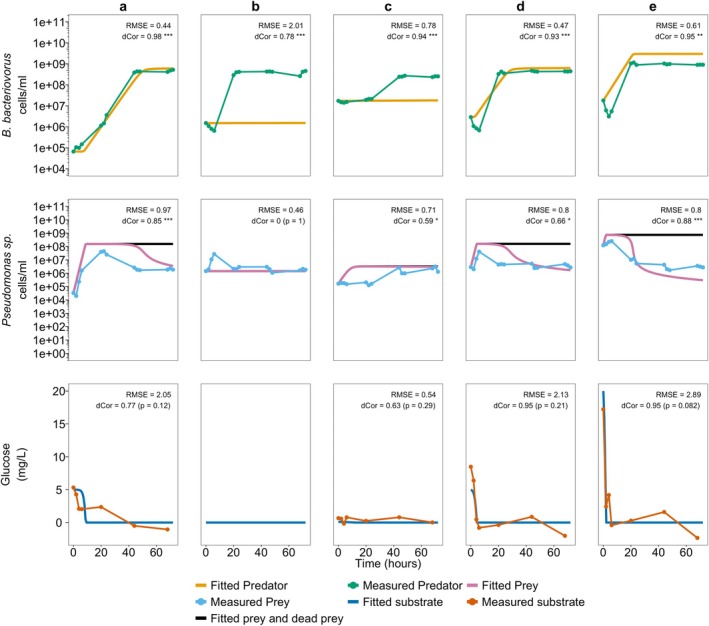
Transient curve of Holling type III (Equations (4), (5), (6)) fitted and experimentally observed *B. bacteriovorus* and *Pseudomonas* sp. (cells/mL) population and glucose (mg/L) during five different batch cultures (a–e) with different initial prey, predator, and glucose substrate concentrations. (a) *X*_0_ = ~10^4^ cells/mL, *Y*_0_ = ~10^4^ cells/mL, *S*_0_ = 5 mg/L. (b) *X*_0_ = ~10^6^ cells/mL, *Y*_0_ = ~10^6^ cells/mL, *S*_0_ = 0 mg/L. (c) *X*_0_ = ~10^5^ cells/mL, *Y*_0_ = ~10^7^ cells/mL, *S*_0_ = 0.1 mg/L. (d) *X*_0_ = ~10^6^ cells/mL, *Y*_0_ = ~10^6^ cells/mL, *S*_0_ = 5 mg/L. (e) *X*_0_ = ~10^8^ cells/mL, *Y*_0_ = ~10^7^ cells/mL, *S*_0_ = 20 mg/L. Raw observed bacterial counts and the model predicted counts are provided in Table S5. Root mean square error (RMSE) and distance correlation (dCor) values for the comparison between the batch model fitted and observed experimental values are shown in the plot. Analysis for the *Pseudomonas* sp. and *B. bacteriovorus* used the log_10_ transformed values. RMSE is expressed in the log_10_(cells/ml). *Statistical significance of dCor: *p < 0.05, **p < 0.01, and ***p < 0.001.

Using these parameters in the batch culture models using Holling type II and Holling type III (Equations (1), (2), (3) and (4), (5), (6), respectively) in MATLAB, we predicted the outcome of a batch system with a range of initial conditions for glucose concentration, predator and prey populations. A summary of the conditions and the predicted and observed system responses is presented in Figure 5 (Holling type II) and Figure 6 (Holling type III) and the raw observed bacterial counts and predicted counts by the model are provided in Table S5. We have superimposed the concentrations of predator and prey measured in the set of batch culture experiments that attempt to recreate the simulated results.

When the initial glucose concentration is higher (5 mg/L and above), the model fits the growth trend of predator (Figure 5a,d,e) which is particularly reflected dCor analysis, where the trends between the model and experimental data being very significantly correlated (*p* < 0.01) in these cases. However, the model does not fit to the prey concentration well after time and the prey concentration declines rapidly to near zero, well below what is observed in experiment.

Notably, when the initial glucose concentration was zero or low (below the half‐saturation constant) the model was not able to capture the dynamics of the predator (Figure 5b,c). In these cases, RMSE analysis showed that for the predator in particular there was a high amount of error between the observed and predicted values and the dCor analysis shows that the model did not follow the trend for neither the prey nor glucose. The model predicted that at these glucose concentrations there would not be any significant growth in the prey population. With the prey population remaining at such a low concentration well below the prey half‐saturation constant, it is predicted by the model that the predator would also not show significant growth. However, in experiment, the *Pseudomonas* sp. prey was still able to grow even in the absence of glucose. As expected, this results in an increase in the predator population.

This is also displayed similarly by the Holling type III model (Figure 6b,c). In contrast, when the initial glucose concentration is higher (5 mg/L and above), the model is able to predict the growth trend of the prey and the predator (Figure 6a,d,e) despite the systematic error present. This is also reflected particularly, in the RMSE analysis, where the error between the observed and predicted values of prey growth is substantially lower in the Holling type III compared to the Holling type II.

### 3.3 Simulations to Explore Different Classes of Population Dynamics in a Chemostat

**FIGURE 7:**
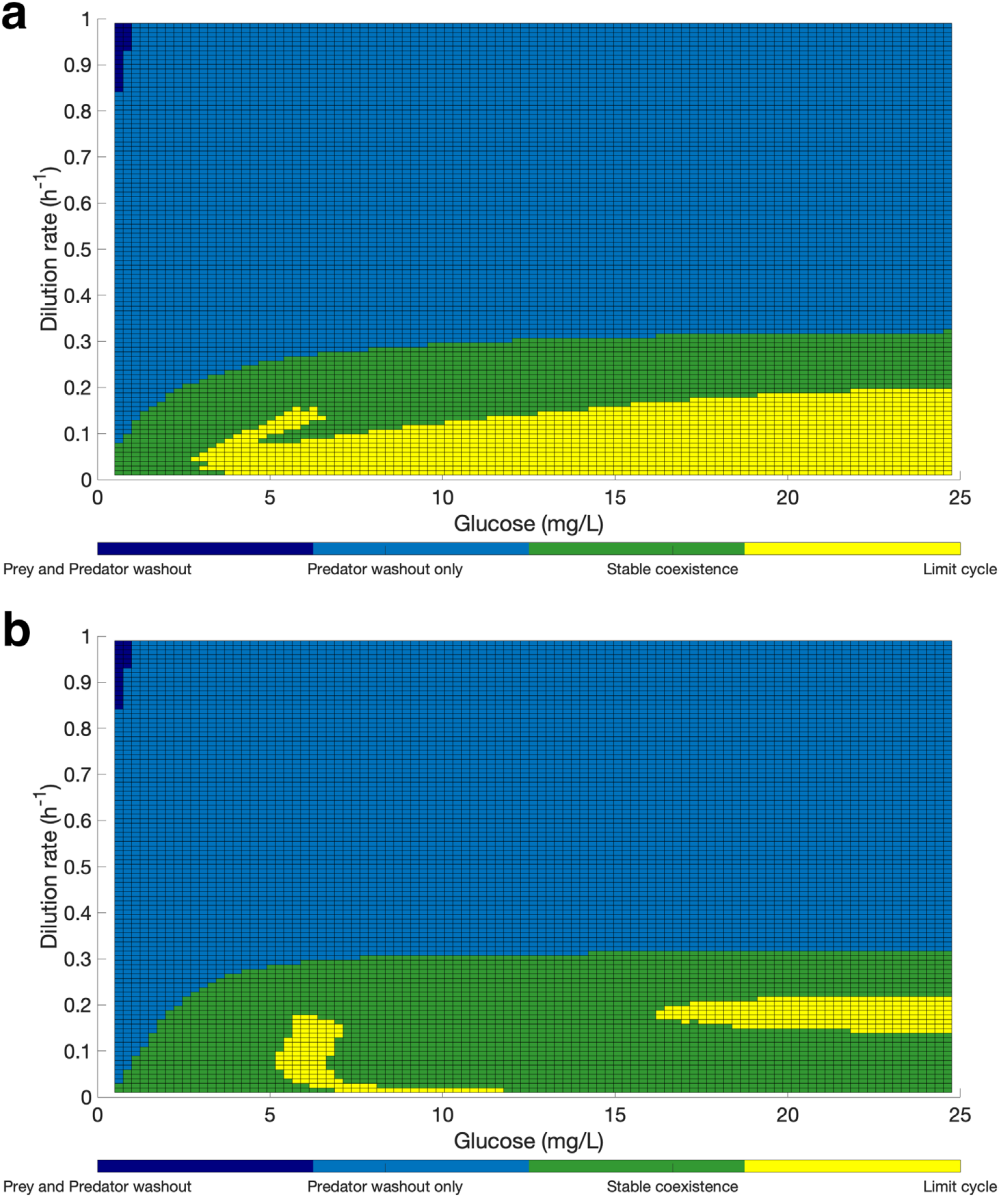
Model predicted surface plot. Displays the different behaviours of *B. bacteriovorus and Pseudomonas* sp. in chemostat culture depending on the glucose in the influent (mg/L) and dilution rate (h^−1^). (a) Holling type II. (b) Holling type III.

The growth kinetic parameters were used to simulate predator prey dynamics using the chemostat culture models using the Holling type II and III response (Equations (7), (8), (9) and (10), (11), (12)). We systematically simulated the dynamics for 100 × 100 plausible combinations of dilution rate and the influent glucose concentration and classified the dynamics using the heuristic rules outlined in the methods above as being either: prey and predator washout, predator washout, stable co‐existence and limit cycle oscillations (Figure 7).

For both models, the outcome of the predator and prey populations is largely determined by the dilution rate of the chemostat, and at the dilution rates higher than 0.4 h^−1^, the predator is washed out regardless of the glucose concentration in the influent. There is a small region in the parameter space, when the dilution rate is really high (approximately above 0.85 h^−1^), and the glucose concentration is low (approximately below 1 mg/L), the prey is also washed out along with the predator.

At a low enough dilution rate and a high enough influent substrate concentration the prey and predator growth are more stable and there is a small parameter space in which there is a limit cycle, regular oscillations in the substrate, predator and prey populations.

**FIGURE 8:**
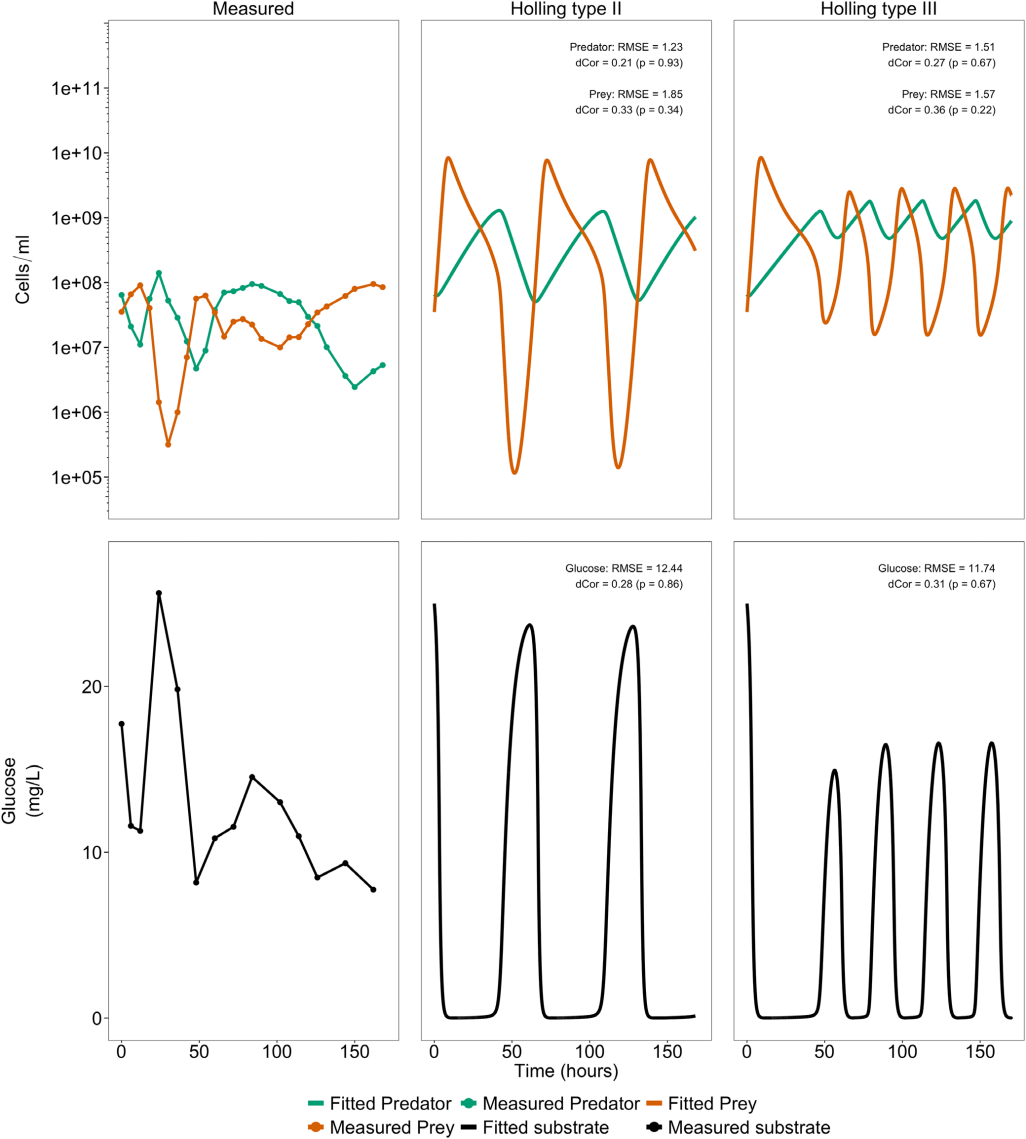
Experimentally observed and model predicted transient responses during chemostat culture for glucose (mg/L), *B. bacteriovorus* and *Pseudomonas* sp. (cells/mL). Initial prey (*X*_0_): 3.5 × 10^7^ cells/mL, initial predator (*Y*_0_): 6.55 × 10^7^ cells/mL, and initial glucose substrate (*S*_0_): 25 mg/L, dilution rate (*D*): 0.17 h^−1^. Root mean square error (RMSE) and distance correlation (dCor) values for the comparison between the chemostat model fitted data and the observed experimental values are shown in the plot. Analysis for the *Pseudomonas* sp. and *B. bacteriovorus* used the log_10_ transformed values. RMSE is expressed in the log_10_(cells/mL). *Statistical significance of dCor: *p < 0.05, **p < 0.01, and ***p < 0.001. Raw observed bacterial counts and the model fitted counts are provided in Table S6.

Figure 8 shows the Holling type II and type III fitted growth of the predator and prey populations under the conditions required to induce oscillations (*X*_0_ = ~10^7^ cells/mL, *Y*_0_ = ~10^7^ cells/mL, *S*_0_ = 25 mg/L, and *D* = 0.17 h^−1^). As expected, *Pseudomonas* sp. prey species grows in response to spikes in glucose, which causes an increase in the predator and subsequent decrease in prey and recovery of glucose in the reactor to continue the cycle. These conditions are recreated experimentally in the chemostat and the abundance of predator and prey through time are recorded. It is apparent that the experimental results are able to confirm some oscillations, and the oscillation of predator and prey are, reassuringly out of phase. However, for both the Holling type II and type III models, the amplitude is an order of magnitude higher than observed and for the Holling type III model, the number of oscillations are also notably longer. These observations are represented in the high RMSE and low dCor values (*p* > 0.05) for the predator, prey, and substrate.

**FIGURE 9:**
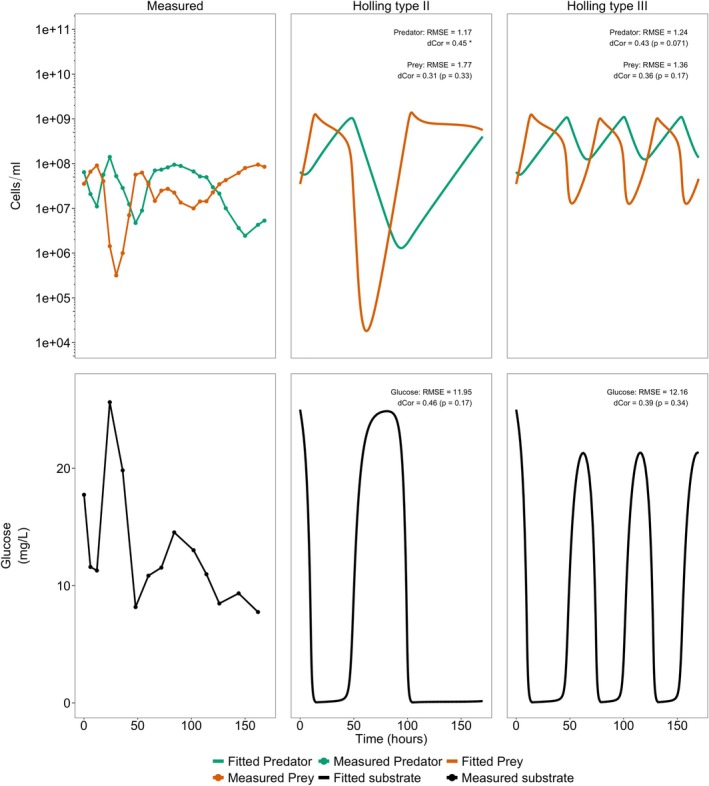
Altered model predicted transient response during chemostat culture for glucose for *B. bacteriovorus* and *Pseudomonas* sp. (cells/mL). Initial prey (*X*_0_): 3.5 × 10^7^ cells/mL, initial predator (*Y*_0_): 6.55 × 10^7^ cells/mL, and initial glucose substrate (*S*_0_): 25 mg/L, dilution rate (*D*): 0.17 h^−1^. Growth parameters used were the same as previous models excluding the maximum prey growth rate: 0.5 h^−1^. Root mean square error (RMSE) and distance correlation (dCor) values for the comparison between the chemostat model fitted data and the observed experimental values are shown in the plot. Analysis for the *Pseudomonas* sp. and *B. bacteriovorus* used the log_10_ transformed values. RMSE is expressed in the log_10_(cells/ml). *Statistical significance of dCor: *p < 0.05, **p < 0.01, and ***p < 0.001. Predicted counts by the model is provided in Table S7.

There are a number of reasons why the experimental chemostat results might deviate from the simulation. It might be that our experimental determination of the model parameters was not completely accurate. To qualitatively assess the likelihood of this, the parameters are subjectively adjusted to see if it could create simulated dynamics that better fit the experimental data. This reduced the simulated cell abundances to values more in line with those in the experiment, the most significant value being the prey maximum growth rate. Reducing this value from 1.0 to 0.5 h^−1^ reduces the maximum population densities predicted by the models. This adjustment also decreased the frequency of oscillations, making the model more comparable to the experimental data (Figure 9), as reflected by a decrease in RMSE and an increase in dCor for both predator and prey. The Holling type III dCor for the predator suggests a moderate correlation and is approaching statistical significance (dCor = 0.424 *p* = 0.07), while the Holling type II dCor is statistically significant (*p* < 0.05) indicating improved agreement between experimental and the model predicted values. However, the model remains imprecise and does not yet fully capture the observed dynamics.

As discussed, the model is not in fact capturing some of the important phenomena that are occurring in the experiment. It may be that the assumption of a mixed planktonic community is a gross oversimplification and that the bacteria are forming slower‐growing biofilms on the walls of the chemostat.

To determine the effect of biofilm growth on the chemostat growth, the experiment was repeated with the similar conditions but instead the biofilm growth on glass slide was obtained and reported below. As before, the model is able to predict the oscillations, but it is otherwise inaccurate. Though there was biofilm found to be growing in the reactor, the total count was similar to the total planktonic bacteria growing in the bulk liquid at 72 h (Table 2) and the total number of bacteria growing in the reactor still did not account for the overestimation shown in the model.

**TABLE 2:** Measurement of the free living planktonic bacterial cells in the bul**k** liquid and the bacterial cells present in biofilm attached to the reactor surface.

Species	State of cells	Number of cells
*B. bacteriovorus*	Planktonic	1.23E+09
*Pseudomonas* sp.		5.57E+10
*B. bacteriovorus*	Biofilm	1.19E+09
*Pseudomonas* sp.		4.03E+10
*B. bacteriovorus*	Planktonic + Biofilm	2.42E+09
*Pseudomonas* sp.		9.60E+10

## 4 Discussion

The objective of this study was to gain a quantitative understanding of a predator–prey system involving a single growth‐limiting nutrient. Unlike traditional optical density or biomass measurements, we used flow cytometry to directly quantify cell concentrations (Ogundero et al. 2022). This approach enabled precise measurement of predator–prey growth.

### 4.1 Experimental Insights Into Predator–Prey Dynamics

*Pseudomonas* sp. growth across a gradient of glucose concentrations was measured and as expected, the relationship followed Monod kinetics (Figure 2), with a maximum growth rate of 1.0 h^−1^ and a half‐saturation constant of 0.18 mg/L. Interestingly, *Pseudomonas* sp. was able to grow in the absence of glucose, which cannot be replicated by a Monod growth equation, which assumes a single limiting resource and thus zero resource means zero growth. Suggesting that, despite best efforts, glucose was not the only growth limiting substrate present. Instead, casein hydrolysate which was provided as a nitrogen source in the media, may have been utilised for growth.

Many studies select yield and half‐saturation constant values from previous literature rather than obtaining them experimentally, which is difficult to do because it requires measurements of glucose at the microgram per litre range (Shirsat et al. 2015). Here, a glucose assay kit enabled us to measure the degradation of the glucose substrate when provided to *Pseudomonas* sp. An average yield of 3.1 × 10^7^ *Pseudomonas* sp. cells/mg of glucose is observed. This yield informs how efficiently glucose is converted into biomass and helps constrain model parameters.

Our findings show that *B. bacteriovorus* growth resembles a sigmoidal numerical response to increasing prey concentrations, consistent with Holling type III kinetics. The *B. bacteriovorus* only show a significant increase in growth rate at prey populations of 10^8^ cells/mL and above. This aligns with evidence that *B. bacteriovorus* is found predominantly in environments of high prey density such as biofilms (Staples and Fry 1973).

This behaviour may result from a combination of traits associated with *B. bacteriovorus* predation. In highly mixed environments such as a chemostat, predator mobility is largely governed by media flow and making directed movement or chemotaxis toward prey unlikely. Instead, *B. bacteriovorus* is thought to rely on speed and frequent random collisions with prey cells to initiate predation (Straley and Conti 1977). At lower prey densities where these collisions are rarer, the trade‐off between energy used to find prey and the energy received from predation for the production of new progeny is no longer favourable. Additionally, as *B. bacteriovorus* cells age without being able to invade a prey cell, the cell becomes slower which further decreases the chance of successful collisions and predation efficiency (Carlson et al. 2020).

However, the reduced predation efficiency as a result of random collision is alone not enough to induce the Holling type III response. Typically an additional mechanism is required such as prey switching or a predator showing a learning process that increases with prey density (DeLong 2021; Morozov and Petrovskii 2013). Alternatively, a more plausible mechanism contributing to *B. bacteriovorus* growth dynamics involves non‐linear interference effects, such as the presence of decoys or inefficient use of prey. For example, at low prey densities, high predator to prey ratios can lead to multiple predators attaching to the same prey cell, ultimately causing detachment without successful invasion (Varon and Shilo 1968). In such cases predator‐attached prey cells and Bdelloplasts (invaded prey cells) may act as decoys reducing the number of viable targets and further limiting predation efficiency under low‐density conditions.

Additionally, if the predator‐to‐prey ratio is sufficiently high, multiple *Bdellovibrio* cells attach to the same prey cell, causing it to burst prematurely before successful invasion can occur (Im et al. 2014). This effect is nonlinear, and at low prey densities, frequent premature lysis events could prevent a significant increase in the predator population. Consequently, as prey density increases, the efficiency of prey conversion to predator biomass may rise disproportionately, contributing to a sigmoidal (Holling type III‐like) numerical response. This could also explain why, at lower prey concentrations, prey populations decline more rapidly than at higher concentrations, where predation initially remains inefficient (Figure 3).

It is important to note in this case that Holling type II may well be appropriate for capturing *B. bacteriovorus* dynamics, as has been shown previously. For example, Hobley et al. (2020) successfully predicted the growth of *B. bacteriovorus* and an *Escherichia coli* prey in batch cultures by using a model with Holling type II dynamics via approximate Bayesian computation (ABC). However, ABC selects the best‐fitting model parameters under specific conditions. In contrast, we undertook a bottom‐up modelling approach that prioritised experimentally derived parameters. This avoids parameter overcompensation, where one parameter can be compensated incorrectly for another during fitting, which can reduce the understanding of intrinsic biological traits, particularly for values such as the half‐saturation constant.

### 4.2 Model Validation and Performance in Batch and Chemostat Systems

Using the batch Holling type II and Holling type III models, we simulated five different batch culture scenarios, with different initial conditions of bacteria and substrate concentrations. Holling type II often overestimated prey loss and underestimated the prey half‐saturation constant. In contrast, Holling type III produced better fits to observed trends when glucose was ≥ 5 mg/L. At concentrations below the glucose half‐saturation constant, both models performed poorly, likely due to growth contributions from casein hydrolysate.

Using the calculated parameters in the chemostat models created with equations in MATLAB (Equations for (7), (8), (9) for Holling type II and Equations (10), (11), (12) for Holling type III), we mapped the combinations of initial conditions for glucose concentration in the influent and, predator and prey population in a chemostat that would lead to a range of different dynamics: prey and predator washout, predator washout, stable co‐existence and limit cycle oscillations. As expected, the potential for predator washout is the largest of the outcomes as *B. bacteriovorus* grows slower than its prey.

The potential for predator prey oscillations is comparably smaller in the chemostat described, which is to be expected as the prey's growth is reliant on one substrate and the action of one predator. Whereas in a natural system with multiple sources of organic substrates and potentially multiple prey and predator species, limit cycles are more commonly observed (Wilkinson 2006). These oscillatory states are particularly valuable for biocontrol applications, where continuous predator re‐inoculation would be impractical. In decentralised systems like drinking water filters, sustained oscillations can maintain predator populations and potentially suppress biofilms more reliably than intermittent chemical dosing. Models such as these, reduce the need for trial‐and‐error experimentation to search for this precise parameter space. Thus, the models increase the speed and efficiency as well as reducing the time and cost associated with all the extra experimentation.

To test the fit of the Holling type II and type III models, a chemostat is run for 168 h with initial conditions that would lead to limit cycle oscillations: initial prey (*X*_0_): 3.5 × 10^7^ cells/mL, initial predator (*Y*_0_): 6.55 × 10^7^ cells/mL, and influent glucose substrate (*S*_0_): 25 mg/L, and dilution rate (*D*): 0.17 h^−1^. As expected, the predator and prey populations exhibit regular oscillations. Though previous studies have theoretically demonstrated this (Hobley et al. 2020; Summers and Kreft 2022)., this study demonstrates one of the few experimentally observed oscillations in *B. bacteriovorus* and their prey (Dulos and Marchand 1984; Varon 1979). One key observation is that when the prey concentration dropped to as low of 3 × 10^5^ cells/mL, the oscillations remained stable which is in agreement with a previous study (Varon et al. 1984) and lower than they had previously theorised would be the required prey concentration needed to maintain stable coexistence (Varon and Zeigler 1978).

### 4.3 Future Directions and Implications for Biocontrol

These results reinforce the utility of dynamic modelling in guiding system design. With the ability to predict oscillatory regions, we can identify optimal operating conditions that minimise chemical use and improve system sustainability. Specifically, both predicting and inducing oscillations could aid in the identification of key ‘low’ points in the prey population where chlorine dosing in drinking water filtration systems could be considered more efficient, maximising the potential to mitigate biofouling while minimising the harmful effects of frequent chlorine usage such as resistance development (Chen et al. 2010; Jin et al. 2020).

Despite this success, our chemostat models could not accurately replicate the regularity of the oscillations as well as the maximum and minimum populations reached. One feature that the batch and chemostat models were unable to capture was the initial decline in the *B. bacteriovorus* population, particularly at high prey densities, where the *B. bacteriovorus* enters the prey cell to form a Bdelloplast and become undetectable by flow cytometry. In this case the inclusion of terms into the model to capture Bdelloplasts and the initial time delay in the predator growth could improve the model as has been previously demonstrated by Hobley et al. (2020), who were able to accurately predict the initial decline in the *B. bacteriovorus population*.

Another limitation is the exclusion of biofilm dynamics. In the second chemostat run, significant *Pseudomonas* sp. biofilm formation was confirmed by quantifying cells on reactor‐placed slides (Table 2). Compared to the planktonic bacteria, bacteria growing in biofilm can consume significant nutrients from the bulk liquid while typically displaying a slower growth rate and is not as easily removed from the reactor by washout (Jass et al. 1995). This biofilm cannot be quantified by sampling from the effluent and can impair model accuracy.

Moreover, the predator *B. bacteriovorus* does not form biofilm but has been shown to be able to move and predate within them (Kadouri and O'Toole 2005; Núñez et al. 2005). This phenomenon was also proposed by Varon et al. (1984), who speculated that the heterogenous biofilm growth present in a bioreactor could present local foci for *B. bacteriovorus* and could be the reason why their model (which assumed evenly distributed planktonic prey growth) overestimated the prey concentration required to maintain *B. bacteriovorus* within the system. This is less of a concern in batch culture studies where there is not a constant supply of nutrients to allow for significant biofilm development and planktonic bacteria remain in the system and are not removed by dilution.

Progeny predatory bacteria that are developing within Bdelloplasts from biofilm forming bacteria may be less likely to be removed from washout than their counterparts in the bulk liquid. Retaining these predators within the system can then lead to a further unexpected decrease of the prey bacteria within the biofilm and the bulk liquid. There is little information on how *B. bacteriovorus* predation is affected by biofilm structure but there have been many studies that have demonstrated that thicker, less porous biofilms prevented the diffusion of antibiotics and limits the predation of bacteriophages to the upper regions of biofilms (Kim et al. 2009).

In order to have a chance of capturing the behaviour in the chemostat, then the equations in the models would have to be augmented with ones that explicitly model the biofilm population, such as in the Freter model (Jones et al. 2003), as well as separate equations for surface attachment, detachment, and diffusion‐limited growth, and it could not be assumed that the growth kinetics of the predator and prey in biofilms would be the same as for planktonic populations (Wang et al. 2015). Indeed, the complexities of biofilms are such that a much more intensive experimental programme would be required.

The structure of biofilms is key when considering how they develop in response to growth limiting nutrients as they are heterogenous and contain pores and channels that allow for diffusion. In thick or compact biofilms there is lower diffusion and less nutrients delivered to bacteria deeper within the biofilms and likewise there is less removal of waste products. These bacteria are slower growing and less metabolically active than both planktonic bacteria and bacteria on the outer surface of the biofilm (Legner et al. 2019). This could also explain why decreasing the growth rate of the prey to 0.5 h^−1^ resulted in better fitting models particularly improving the Holling type II fit (dCor = 0.45, *p* < 0.05) (Figure 9).

These results support the use Holling type II under conditions of constant nutrient availability and waste removal, where prey is often in the exponential phase and readily vulnerable to predation. Here, predation is more efficient at lower prey densities and saturates smoothly, which are traits consistent with the Holling type II model. Reducing the prey growth rate then significantly improves the model fit as it allows more time for predation rate to reach saturation. Whereas in the batch system, after time, the bacteria are less metabolically active and at lower prey densities, predation efficiency increases non‐linearly which is not as well captured by Holling type II.

In conclusion, the study utilised Holling type II and Holling type III models of nonlinear ordinary differential equations to express the interaction between *B. bacteriovorus* predators, *Pseudomonas* sp. prey and glucose. With experimentally obtained growth parameters; the models were used to predict the dynamics of *B. bacteriovorus* predation in batch cultures. The prey bacteria growth on a substrate adheres to Monod kinetics and the *B. bacteriovorus* predator growth on prey adheres to both Holling models but best to the Holling type III numerical response. With these same parameters, both the Holling type II and the Holling type III models were able to predict the conditions needed to cause different system behaviours including predator prey oscillations. Oscillations were induced experimentally in the chemostat, but biofilm growth, which was found to be significant within the continuous system is likely to have a profound effect on the accuracy of the models. Capturing these dynamics in a verifiable, validated model would require a substantive experimental programme. Nonetheless, the ability to induce predator prey oscillations is vital in the application of *B. bacteriovorus* as a self‐sustainable biocontrol.

## Author Contributions

**Ayo Ogundero:** conceptualization, investigation, writing – original draft, writing – review and editing, data curation. **Stephanie Connelly:** conceptualization, investigation, writing – review and editing, supervision, data curation. **William T. Sloan:** conceptualization, investigation, writing – review and editing, supervision, data curation.

## Conflicts of Interest

The authors declare no conflicts of interest.

## Supporting information


**TABLE S1:** The growth of *Psedumonas* on different concentrations of glucose.

**TABLE S2:** The growth kinetics of *Psedumonas* on different concentrations of glucose. Type describes whether the data is *M* (measured) or *F* (fitted) using the Monod equation. *U* = growth rate; UMAX = maximum growth rate; *K*_s_ = saturation constant.

**TABLE S3:** The growth of *Bdellovirbio* on different concentrations of *Pseudmonas* sp. (prey).

**TABLE S4:** The growth rate of *Bdellovirbio* on different concentrations of Pseudmonas sp. (prey). Type describes whether the data is measured by experiment (*M*) or fitted using the Monod equation (*F*_*M*) or the Holling III equation (*F*_*H*). U = growth rate.

**TABLE S5:** The growth and changes in concentration of predator, prey, and substrate in five different batches. CELL describes the object measured: PREY (prey), PRED (predator), and SUB (substrate). msubstrate is the concentration of the substrate (mg/L). Type describes whether the data is measured by experiment (expPREY, expPRED, and expSUB) or whether its data built in the model (mprey, mpred, and msub). mdead is the concentration of prey predicted by the model to be dead. Model indicates the model used: H2 = Holling type II and H3 = Holling type III.

**TABLE S6:** The growth and changes in concentration of predator, prey, and substrate in a chemostat. ID describes the object measured: CELL (prey or predatory), and SUB (substrate). msubstrate is the concentration of the substrate (mg/L). Type and name describes whether the data is measured by experiment (EXP) (expPREY, expPRED, and expSUB) or whether it its data built in the model (MOD_H2 for Holling type II and MOD_H3 for Holling type III) (mprey, mpred, and msub).

**TABLE S7:** The growth and changes in concentration of predator, prey, and substrate in chemostat as predicted by the altered chemostat model. Growth parameters used were the same as previous models excluding the maximum prey growth rate: 0.5 h^−1^. ID describes the object measured: CELL (prey or predatory), and SUB (substrate). msubstrate is the concentration of the substrate (mg/L). Type and name describes whether the data is measured by experiment (EXP) (expPREY, expPRED, and expSUB) or whether it its data built in the model (MOD_H2 for Holling type II and MOD_H3 for Holling type III) (mprey, mpred, and msub).

**APPENDIX S1:** Supporting information.

## Data Availability

The data that supports the findings of this study are available in the supplementary material of this article.
