# Supplementary material for: Developing Lotka–Volterra Based Models to Describe Bdellovibrio Predation in a Batch and Chemostat Experimental System
Source: Environ Microbiol Rep. 2025 Jul 6;17(4):e70141. doi: 10.1111/1758-2229.70141 (PMC12229818; doi:10.1111/1758-2229.70141)
Supplement: Supplementary file 8 — APPENDIX S1: Supporting information. [file EMI4-17-e70141-s007.docx]

Supporting information

### MATLAB code for batch predator prey model

%Holling III batch

%The code for the Holling II batch is the same as this but with a change in the appropiate model parameters (mu_pred and ksat_pred) and the equation for the predator specific growth rate;m_pred = mu_pred * X / (k2 + X);

function simple_batch

% Time range for model output

timerange = linspace(0, 72, 100); % time in hours

% Parameters

mu_prey = 1.0; % max growth rate of prey (1/h)

mu_pred = 0.244; % max growth rate of predator (1/h)

k_S = 0.18; % half-sat. const. for substrate (mg/L)

k2 = 2.74e15; % half-sat. const. for predator (cells^2/ml^2)

eta_x = 1.95; % predator yield on prey (predator cells per 1 prey cell)

eta_S = 3.1e7; % prey yield on substrate (prey cells per mg substrate)

% Initial conditions

x0 = 1e6; % prey (cells/ml)

y0 = 1e6; % predator (cells/ml)

S0 = 5; % substrate (mg/L)

z0 = 0; % dead prey (cells/ml)

a0 = [x0; y0; z0; S0]; % state variable vector

% Solve ODEs

[t, a] = ode45(@(t, a) differentials(t, a, mu_prey, mu_pred, k2, eta_x, eta_S, k_S), timerange, a0);

% Save to CSV

writematrix([t, a(:,1), a(:,2), a(:,3) + a(:,1), a(:,4)], 'batchmodel_output.csv');

% Plotting

figure(1)

tiledlayout(4, 1)

nexttile

plot(t, a(:,1), 'b', t, a(:,2), 'r', t, a(:,3)+a(:,1), 'k')

set(gca, 'YScale', 'log')

xlabel('Time (hours)')

ylabel('Log₁₀(Number)')

legend('Prey', 'Predator', 'Prey + Dead Prey')

nexttile

plot(t, a(:,2), 'r')

set(gca, 'YScale', 'log')

xlabel('Time (hours)'), ylabel('Predator Log₁₀(cells/ml)')

legend('Predator')

nexttile

plot(t, a(:,1), 'b')

set(gca, 'YScale', 'log')

xlabel('Time (hours)'), ylabel('Prey Log₁₀(cells/ml)')

legend('Prey')

nexttile

plot(t, a(:,4), 'g')

xlabel('Time (hours)'), ylabel('Substrate (mg/L)')

legend('Substrate')

end

function dadt = differentials(~, a, mu_prey, mu_pred, k2, eta_x, eta_S, k_S)

% State variables

X = a(1); % prey

Y = a(2); % predator

Z = a(3); % dead prey

S = a(4); % substrate

% Specific growth rates

m_prey = mu_prey * S / (k_S + S);

m_pred = mu_pred * X^2 / (k2 + X^2);

% ODEs

dadt = zeros(4,1);

dadt(1) = m_prey * X - m_pred * Y / eta_x; % prey

dadt(2) = m_pred * Y; % predator

dadt(3) = m_pred * Y / eta_x; % dead prey

dadt(4) = -m_prey * X / eta_S; % substrate

end

### MATLAB code for chemostat parameter simulations

%Holling III chemostat parameter simulations

%The code for the Holling II chemostat is the same as this but with a change in the appropiate model parameters (mu_pred and ksat_pred) and the equation for the predator specific growth rate; m_pred = mu_pred*a(1)/(k2 + a(1));

function chemostat_predator_Li_Kuang_search

% Parameters

mu_prey = 1.0; % max growth rate of prey (1/h)

mu_pred = 0.244; % max growth rate of predator (1/h)

ksat_S = 0.18; % half-sat. const. for substrate (mg/L)

ksat_pred = 2.74e15; % half-sat. const. for predator (cells^2/ml^2)

eta_x = 1.95; % predator yield on prey (predator cells per 1 prey cell)

eta_S = 3.1e7; % prey yield on substrate (prey cells per mg substrate)

S0 = 25; % Initial substrate concentration mg/L

n = 40 %run the model for approx n retention times

%Define state variables

% State variable 1 (a(:,1)) is the prey (cells/ml)

% State variable 2 (a(:,2)) is the predator (cells/ml)

% State variable 3 (a(:,3)) is the influent substrate (mg/L)

a0 = [ 1*10^7 1*10^7 S0]

num = 100 %The number of points along each axis (D and S0).

Dmin = 0.01 % minimum diluation rate (1/h)

Dmax = 1.0 % maximum diluation rate (1/h)

Smin = 0.5 % minimum influent substrate (mg/L)

Smax = 25 % minimum influent substrate (mg/L)

tol = 1e3

tol2 = 1e-3

tol3 = 1e7

for i = 1:num

i

D = Dmin + (Dmax - Dmin)*(i-1)/num;

if D > 0.179

D;

end

for j = 1:num

j;

S0 = Smin + (Smax - Smin)*(j-1)/num;

if S0 >19.9

S0;

end

X(i,j) = S0;

Y(i,j) = D;

a0 = [ 1*10^7 10^7 S0];

tau = [0 n/D];

[t,a] = ode45(@(t,a) differentials(t,a,D,S0,mu_prey, mu_pred, ksat_pred, eta_x, eta_S, ksat_S),tau,a0);

len = length(t);

if a(len,3) > S0-tol2

%washout

Z(i,j) = 1;

elseif ( a(len,1) > tol ) && (a(len,2) < tol)

Z(i,j) = 2;

else

% there is a non trivial solution and we want to find out if it

% oscilates or not. Heuristically an ocilattion solution will

% pass through its meadian value a few times in the later hald

% of the solution

med = median(a(round(len/2):len,1));

dif = a(round(len/2):len,1) -med;

if std(dif) > tol3

sgn = sign(dif);

%count the number of times we cut through the median

cnt = 0;

for ii = 4:length(sgn)

if ( sgn(ii-3)+sgn(ii-2))*( sgn(ii-1) + sgn(ii)) <0

cnt = cnt + 1;

end

end

if cnt < 4

Z(i,j) = 3;

else

Z(i,j) = 4;

end

else

Z(i,j) = 3;

end

end

end

end

% Create surface plot

figure(1)

s = surface(X, Y, Z);

custom_colors = [

0.0, 0.0, 0.5; % dark blue

0.0, 0.45, 0.74; % blue

0.2, 0.6, 0.2; % green

1.0, 1.0, 0.0 % yellow

];

colormap(custom_colors)

c = colorbar('southoutside'); % places the colorbar below the x-axis

set(c, 'Ticks', [1, 2, 3, 4], ...

'TickLabels', {'Prey and Predator washout', 'Predator washout only', 'Stable coexistence', 'Limit cycle'}, ...

'FontSize', 20)

xlabel('Glucose (mg/L)', 'FontSize', 30)

ylabel('Dilution rate (h^{-1})', 'FontSize', 30)

set(gca, 'FontSize', 26)

set(gcf, 'Color', 'w')

saveas(gcf, 'test_splot.tiff', 'tiff')

function dadt = differentials(t,a,D,S0,mu_prey, mu_pred, k2, eta_x, eta_S, k_S)

% Specific growth rates

m_prey = (mu_prey)*a(3)/(k_S + a(3));

m_pred = mu_pred*a(1)^2/(k2 + a(1)^2);

% ODEs

dadt = zeros(size(a));

dadt(1) = -D*a(1) + m_prey*a(1) - m_pred*a(2)/eta_x ;

dadt(2) = -D*a(2)+ m_pred*a(2);

dadt(3) = D*(S0-a(3))-(m_prey)*a(1)/eta_S ;

if (a(1) + dadt(1)) < 0

dadt(1) = -a(1);

a(1) = 0.0;

end

if (a(2) + dadt(2)) < 0

dadt(2) = -a(2);

a(2) = 0.0;

end

if (a(3) + dadt(3)) < 0

dadt(3) = -a(3);

a(3) = 0.0;

end

### MATLAB code for chemostat predator prey model

%Holling III chemostat

%The code for the Holling II chemostat is the same as this but with a change in the appropiate model parameters (mu_pred and ksat_pred) and the equation for the predator specific growth rate; m_pred = mu_pred*a(1)/(k2 + a(1));

function chemostat_predator

%legend

% s0 is the influent substrate concentration (mg/L)

% x0 is the initial prey concentration (cells/ml)

% y0 is the initial predator concentration (cells/ml)

% eta_x is the yield of predator consuming prey (predator cells per 1 prey cell)

% eta_S is the yield of prey feeding on substrate (prey cells per mg substrate)

% mu_prey = maximum growth rate of prey feeding on substrate (h-1)

% ksat_S is half saturation constant for prey feeding on substrate (mg/L)

% mu_pred = max growth rate of predator (h-1)

% ksat_pred = half saturation constant of predator feeding on prey (cells2/ml2)

% D is the diluation rate (h-1)

%model parameters - these values could be estimated in batch experiments.

S0 = 25;

eta_S = 3.1*10^7;

eta_x = 1.95;

D = 0.17

mu_prey = 1.0;

mu_pred = 0.244;

ksat_S = 0.18;

ksat_pred = 2.74*10^15;

n = 40 %run the model for approx n retention times

tau = [0 n/D]; %

%Initial conditions

Start_S = S0

Start_prey = 3.5*10^7

Start_pred = 6.55*10^7

%Define state variables

a0 = [ Start_prey Start_pred Start_S ]

% State variable 1 (a(:,1)) is the prey (cells/ml)

% State variable 2 (a(:,2)) is the predator (cells/ml)

% State variable 3 (a(:,3)) is the substrate (mg/L)

%Define function

[t,a] = ode45(@(t,a) differentials(t,a,D,S0,mu_prey, mu_pred, ksat_pred, eta_x, eta_S, ksat_S),tau,a0);

%transfer data to csv

writematrix([t(:),a(:,1),a(:,2),a(:,3)], 'chemostatmodel_output.csv');

%Viewing the result

figure(1)

plot(t,a(:,1))

set(gca, 'YScale', 'log')

hold on

plot(t,a(:,2))

set(gca, 'YScale', 'log')

plot(t,a(:,3))

legend('Prey', 'Predator','Substrate' )

hold off

%Then tau - the real time

tt = t;

S = a(:,3);

x = a(:,1);

y = a(:,2);

figure(2)

plot(tt,S);

title('Substrate unscaled')

figure(3)

plot(tt,x);

set(gca, 'YScale', 'log')

title('Prey unscaled')

figure(4)

plot(tt,y);

set(gca, 'YScale', 'log')

title('Predator unscaled')

figure(5)

plot(x,y)

function dadt = differentials(t,a,D,S0,mu_prey, mu_pred, k2, eta_x, eta_S, k_S)

% Specific growth rates

m_prey = (mu_prey)*a(3)/(k_S + a(3));

m_pred = mu_pred*a(1)^2/(k2 + a(1)^2);

% ODEs

dadt = zeros(size(a));

dadt(1) = -D*a(1) + m_prey*a(1) - m_pred*a(2)/eta_x ;

dadt(2) = -D*a(2)+ m_pred*a(2);

dadt(3) = D*(S0-a(3))-(m_prey)*a(1)/eta_S ;

if (a(1) + dadt(1)) < 0

dadt(1) = -a(1);

a(1) = 0.0;

end

if (a(2) + dadt(2)) < 0

dadt(2) = -a(2);

a(2) = 0.0;

end

if (a(3) + dadt(3)) < 0

dadt(3) = -a(3);

a(3) = 0.0;

end
